# Challenges to optimising uptake and delivery of a HPV vaccination programme for men who have sex with men

**DOI:** 10.1080/21645515.2018.1560783

**Published:** 2019-01-30

**Authors:** Alice S. Forster, Richard Gilson

**Affiliations:** a Research Department of Behavioural Science and Health, UCL, London, UK; b UCL Centre for Clinical Research in Infection and Sexual Health, The Mortimer Market Centre, UCL, London, UK

**Keywords:** Papillomavirus vaccines, review, sexual and gender minorities

## Abstract

Human papillomavirus (HPV) vaccine programmes targeted at men who have sex with men (MSM) may reduce HPV-related disease burden among this at-risk group in countries where uptake of the vaccine among adolescent girls is sub-optimal and where adolescent boys are not routinely vaccinated. There are challenges to optimising the impact of a MSM programme: ensuring good uptake, understanding the effectiveness of the vaccine in this population and considering the longevity of the programme. Furthermore, monitoring of uptake and ensuring that delivery of the programme does not deprive other aspects of sexual health service resources may present challenges to programme evaluation and delivery. We draw on experience from the UK HPV vaccination programme for MSM, delivered in sexual health and HIV clinics, to better understand these challenges with the aim of supporting the implementation of similar programmes elsewhere in the world.

Every year in the UK, human papillomavirus (HPV) causes approximately 500 oropharyngeal, 300 anal and 200 penile cancers in men^1^ and men who have sex with men (MSM) are disproportionally burdened by diseases attributable to this infection.^2^ Furthermore, around 44,000 of the estimated 515,000 MSM in the UK are living with HIV^3,4^ and these men are 38 times more likely to develop anal cancer compared to HIV-negative men.^5^ People living with HIV often present at a later stage of disease and with more aggressive tumours.^6,7^ Controlling HPV infections will significantly reduce cancer incidence in men living with HIV and HIV-negative men.

As of March 2017, almost half of all countries globally had HPV vaccine programmes in place or in planning for adolescent girls, however fewer countries had organised programmes for adolescent boys.^8^ While good uptake of HPV vaccination among girls will provide indirect protection to boys from HPV-related diseases, it will likely confer little, if any, benefit to MSM.^9,10^ This has the potential to widen disparity in the burden of HPV-related disease among MSM compared to the heterosexual community. In response to this, and in the absence of an adolescent boys HPV vaccination programme, the UK health departments introduced HPV vaccination for MSM. At first the vaccine was delivered as part of a pilot in England from 2016, and later expanded to a full programme across the UK, delivered via sexual health and HIV clinics. The Gardasil® (Merck) HPV vaccine is offered opportunistically to MSM attending these clinics who are ≤45 years old. A three-dose schedule is being used, aiming for all doses to be delivered within 12 months, although 24 months is considered clinically acceptable.^11^ While this programme represents an opportunity to reduce inequalities in HPV-related disease, there are several challenges to delivering this programme and optimising its impact, which will be the focus of this commentary.

## Challenges to optimising the impact of the vaccine programme

### Maximising uptake of the vaccine

Good uptake of the vaccine is critical to the success of the programme. Around 46% of eligible men received the first dose of the vaccine during the first year of the pilot in participating clinics.^11^ Of these men, 43% received the second dose and 6% received the third dose. The course completion rate will have been underestimated, as completing the series within 24 months is considered acceptable. Additionally, many of the initial issues with coding that occurred as the pilot was implemented should now be resolved, although some may still exist. Although uptake is likely to improve as the programme becomes established, there is still a need for research to better understand why men are not receiving the vaccine or completing the series, and subsequently to develop and test interventions to improve uptake.

We have some understanding of why uptake may be incomplete. Although awareness of HPV vaccination is likely to be low among MSM,^12^ data from the US show that those who receive a recommendation for vaccination from a health professional are more likely to be vaccinated than those who do not.^13,14^ However, only men who disclose their sexual orientation/behaviour to a health professional will receive such a recommendation. Indeed, disclosure of sexual orientation to a health professional is associated with acceptability of receiving the vaccine.^15,16^ Some men report feeling uncomfortable discussing their sexual orientation/identity/behaviour with a health professional^17^ and around 26% of MSM had not disclosed their sexual orientation to a health professional (although this sample was not representative of the population so the percentage disclosing may vary on a national level).^16^ Furthermore, even when disclosure is made, it is likely to occur many years after first sexual contact with a man.^15,16^ However, the barriers to disclosure are potentially modifiable, for example by making healthcare environments welcoming to individuals of all sexual orientations.^18^ The MSM programme in the UK is only delivered via sexual health and HIV clinics, which are adept at working with individuals of all orientations, so issues of disclosure may be less of a problem than if the vaccine was being offered in venues less used to discussing sexual orientation.

Perhaps unsurprisingly, men with positive health beliefs about the vaccine are more likely to intend to get vaccinated,^14,16,19^ for example perceiving that they are vulnerable to a HPV infection. We also know that some MSM are concerned about stigma associated with an ‘MSM vaccine’.^20^ However, there is little evidence that interventions that aim to change individuals’ health beliefs improve vaccination uptake^21^ and further work is needed to develop and test alternative approaches to improving uptake in this group.

### Vaccine efficacy

A further challenge to optimising the impact of the programme is the question of how efficacious the vaccine is in this population. The available vaccines work best prior to contact with the virus.^22,23^ Estimates suggest that around 28% of young women acquire a HPV infection within a year of initiating sexual activity, rising to 62% within four years^24^ and this is the rationale for the target age of the adolescent girls programme. However, it is likely that the majority of MSM will only disclose their sexual orientation to a health professional many years after first sexual contact with a man,^15,16^ making it likely that a programme that relies on MSM declaring their sexuality will not provide optimal protection to these men. Nonetheless, the MSM programme was deemed cost-effective in the UK, taking into account the prevalence of HPV infection in the target population. The vaccine will still provide protection against the HPV types covered in the vaccine that men are not currently infected with, as well as preventing re-infection with types men have previously come into contact with.

## Challenges to delivering the programme

### Monitoring of uptake

A key metric of success of the programme is uptake, and this needs to be monitored carefully. Individuals may present to any sexual health clinic in the UK. However, sexual health clinic attendance is confidential outside of the individual’s immediate care team, so it is not possible to link attendances between clinics in the data that is collected nationally to monitor clinical service activity. For this reason, HPV vaccine uptake figures are likely to be inaccurate to some degree. For example, an individual attending a clinic may be ineligible for vaccination due to having received it elsewhere. This could particularly be a problem in the capital city, London, where there are multiple clinics that are geographically close and where there is a large MSM population. However, data from the pilot suggest that the majority of men who have received one dose of the HPV vaccine preferred to have subsequent doses at the same clinic,^11^ suggesting that it will be possible to monitor second and third dose uptake with reasonable accuracy. It may also be possible to assess uptake by measuring HPV antibody levels in unlinked anonymous blood samples from syphilis testing.

### Ensuring that delivery of the programme does not deprive other aspects of sexual health service resources

The introduction of a new, free-at-the-point-of-receipt, cancer-preventing vaccine in sexual health services has the potential to add considerable burden with increased numbers of men accessing services. To explore if this was the case, Public Health England who managed the pilot programme surveyed attendees about their motivation for attending the sexual health or HIV clinic. Around 8% were first time attendees and only 10% of these attended primarily to get the vaccine.^11^ Furthermore, electronic patient records of attendances did not show any large increases following the start of the pilot, although data were limited to the first nine months of the pilot.^11^ It is unlikely that this opportunistic programme will overburden sexual health services.

### The longevity of the programme

In July 2018, the health departments in England, Scotland and Wales announced that adolescent boys would be added to the HPV immunisation programme. This may shorten the lifespan of the MSM programme. MSM vaccinated via the adolescent boys programme will not need vaccination when they start to come into contact with sexual health services. However, men up to age 45 are eligible for the vaccine as part of the MSM programme so it will be many years before there are no eligible men. Furthermore, there will always be those who missed the adolescent boys programme, including those who moved to the UK after the age of 12–13. There will, however, likely be a re-evaluation of cost-effectiveness once the pool of MSM vaccinated as adolescents increases.

## Conclusions

We present learning from the implementation of a national HPV vaccination programme specifically for MSM. Where uptake of HPV vaccination among adolescent girls is sub-optimal and where boys are not routinely offered the vaccine, there may be an equity and cost-effectiveness argument for a MSM programme. Other countries can be reassured that there has not been a surge in demand on sexual health services for the vaccine, but they may need to increase efforts to maximise vaccination initiation and completion. Furthermore, it will be important that processes are in place to enable a robust evaluation of implementation. Such a programme may not always be cost-effective given that such decisions are made on a national basis and will also vary according to the threshold for willingness to pay. Decisions regarding the longevity of the programme will need to consider wider policy changes that impact the programme. The early experience of the UK programme can offer guidance to other countries to help maximise uptake and enhance delivery.
